# Development and Validation of a Prediction Score for Complications after Hepatectomy in Hepatitis B-Related Hepatocellular Carcinoma Patients

**DOI:** 10.1371/journal.pone.0105114

**Published:** 2014-08-15

**Authors:** Haiqing Wang, Jian Yang, Jiayin Yang, Li Jiang, Tianfu Wen, Wentao Wang, Mingqing Xu, Bo Li, Lunan Yan

**Affiliations:** Department of Liver Surgery, West China Hospital of Sichuan University, Chengdu, Sichuan Province, China; The University of Hong Kong, Hong Kong

## Abstract

**Objective and Background:**

The aim of the present study was to develop and validate a prediction score for postoperative complications by severity and guide perioperative management and patient selection in hepatitis B-related hepatocellular carcinoma patients undergoing liver resection.

**Methods:**

A total of 1543 consecutive liver resections cases were included in the study. Randomly selected sample set of 70% of the study cohort was used to develop a score to predict complications III–V and the remaining 30% was used to validate the score. Based on the preoperative and predictable intraoperative parameters, logistic regression analysis was used to identify risk factors and create an integer score for the predicting of complication.

**Results:**

American Society of Anesthesiologists category, portal hypertension, major liver resection (more than 3 segments) and extrahepatic procedures were identified as independent predictors for complications III–V by logistic regression analysis. A score system integrating these 4 factors was stratified into three groups and significantly predicted the risk of complications III–V, with a rate of 1.6%, 11.9% and 65.6% for low, moderate and high risk, respectively. Using the score, the complications risk could be predicted accurately in the validation set, without significant differences between predicted (10.4%) and observed (8.4%) risks for complications III–V (P = 0.466).

**Conclusions:**

Based on four preoperative risk factors, we have developed and validated an integer-based risk score to predict postoperative severe complications after liver resection for hepatitis B-related hepatocellular carcinoma patients in high-volume surgical center. This score may contribute to preoperative risk stratification and clinical decision-making.

## Introduction

With the refinement of surgical techniques and perioperative management in liver surgery in the last decades,postoperative morbidity and mortality has markedly decreased. According to several studies with large sample, the reported mortality after liver resection is less than 4% [1]–[4], however, the risk of postoperative complication remains high, with the incidence ranging from 20% to 50% [1], [3], [5]. Therefore, the focus of liver surgery has turned on strategies to prevent nonlethal complications and develop tools to identify preoperatively potential patients at higher risk for severe complications [1]. Many factors may contribute to postoperative complications and have been verified by other studies [1], [2], [6]–[14], including liver function, portal hypertension, extent of liver resection,blood loss and anesthesiologists category and so on. To prevent complications,it is essential to identify, ideally preoperatively, those patients at risk to develop poor outcome and perform prevention strategies [14]. A simple and readily available prediction score to comprehensively identify patients undergoing liver resection at risk for postoperative severe complications is necessary and urgent. In addition, to enable meaningful protective interventions initiated before surgery or plan the operation, only predictive model including preoperative and predictable intraoperative parameters would perform better [1].

There have been a variety of predictive models developed to stratify risk patients undergoing liver resection [1], [2], [8]–[12]. For example, Breitenstein [1] developed and validated a simple score based on preoperative parameters to predict postoperative complications by severity after liver resection. Although with importance, these studies included liver resections with various diseases and could not be applicable to hepatitis B (HBV)-related hepatocellular carcinoma (HCC) patients because of the abnormalities of the liver parenchyma. As we all known, 54% of HCCs occurred in China and 80% of cases were attributable to chronic hepatitis B viral infections [15], [16]. So, the aim was to develop and validate a simple score to stratify patients preoperatively into risk categories for procedural complications in hepatitis B-related HCC patients undergoing liver resection.

## Materials and Methods

### Study Design and Population

Between January 2009 and March 2013, 1543 consecutive liver resections for HBV-related HCC were included in our study. All the patients were diagnosed with HCC proved by histology and with HBV infection or a history of HBV infection. The selection criteria for hepatecotmy was as follows: (1) Only patients with the Child-Turcotte-Pugh (CTP) score A were considered for hepatectomy in our center to prevent from poor outcomes, (2) The estimated remnant liver volume was more than 50% of the total functional liver volume, (3) HCC patients without metastasis. Patients undergoing emergency surgery were excluded. According to the severity of postoperative complications, the cohort of 1543 patients was divided into two groups to identify risk factors for postoperative complications. One group was with no complication or only complications grades I to II (control group) and the other group was with complications grades III to V (severe complication group). To develop and validate a predictive score for complication stratification, the cohort was randomly divided into the development group (randomly selected 70% of the cohort) and the validation group (the remaining 30%).

### Ethics Statement

Records for patients data of pre-, intra-, and postoperative parameters, and postoperative outcomes were collected prospectively in the West China Hospital of Liver Cancer Registry Database. All of the patients had provided written and valid informed consent before surgical operation. The protocol was approved by the Medical Ethics Committee of West China Hospital, Sichuan University, and the study protocol was carried out in accordance with the Declaration of Helsinki.

### Perioperative Management

All the included patients underwent a thorough case history enquiry, physical examination and routine preoperative laboratory measurements. Echocardiography, chest radiography or computed tomography, pulmonary function test and coronary angiography were carried out if necessary. Preoperative imaging examinations to evaluate the tumor included contrast computed tomography or magnetic resonance imaging of the abdomen. American Society of Anesthesiologists (ASA) category was used for anesthetic assessment. Patients were explored through an extended right subcostal incision and intraoperative ultrasonography was performed routinely. Hemihepatic vascular inflow occlusion [17] or Pringle maneuver [18] were used according to the surgeon's preference. Liver parenchyma division was performed using the Hooking ligation technique [17], [19], [20]or an ultrasonic dissector.

### Definition of the Complications and Parameters

The Clavien-Dindo complications classification system [21], [22] was used for postoperative complications grading. Complications grades of III–V were considered as severe complication and defined as the outcome factor for the development of the present prediction score. The 50–50 criteria [7], defined as prothrombin time<50% and serum bilirubin level>50µmol/L on the day 5 after liver resection, was defined as liver failure. Portal hypertension was defined as esophageal varices detected by endoscopy or a splenomegaly (major diameter >12 cm) with a platelet count <100 000/mm^3^ according to the Barcelona Clinic Liver Cancer group criteria [23]. Mortality were defined as death within 30 days after surgery or death before discharge involving a hospital stay >30 days. For individual preexisting disease, we used a modificatory Charlson index [24], [25] to quantify comorbidities. Since all patients had cancer and HBV infection, the two comorbidities were not included in the calculation. Since severe liver disease such as portal hypertension was very common and crucial for postoperative complications [23], [26], portal hypertension was considered as an independent risk factor to analyze and was removed from the Charlson index. Liver resection with more than 3 segments was defined as major resection, or as minor resection. Extrahepatic procedures included all other operations, except liver resection, such as bowel resection, adrenalectomy, diaphragm resection, biliary tract exploration and adhesion separation due to reoperation.

### Selection of Predictive Parameters and Strategy for Score Development

We followed the standard approach to develop and validate a prediction score [1], [2], [13], [14], [27], [28]. We first compared the pre-, intra-, and postoperative clinical features between complication 0–II group and complication III–V group by univariate analysis. Only preoperative and predictable intraoperative variables with significant difference on univariate analysis were considered for the development of prediction model. We randomly selected 70% of the whole cohort as the score development group (1080 patients). Significant variables in univariate analysis were chosen for multivariate logistic regression (FSTEP method) in the development group to evaluate their independent predictive value for complication III–V [12]. The regression coefficients were transformed into integer-based weighted point system for stratifying complication III–V risk. The reference for each variable was assigned a value of zero, while the variables were assigned numerical value as 0 to 3 based on the beta coefficient. Afterward, the points for each predictor were added to get the individual risk. The risk scores were stratified as follows: (1) low risk, 0 to 3; (2) moderate risk, 4 to 6; (3) high risk, more than 7, based on their numerical values.

At last, the remaining 30% sample was used for the validation group to ensure the predictive risk. We calibrated the multivariate logistic regression model by comparing the predictive risk with the mean observed risk for complications grades III to V in the validation groups [1].

### Statistical Analysis

Statistical analysis was performed using SPSS Version 17 statistical analysis software. The Student *t*-test and Mann-Whitney U tests were used to compare continuous variables when appropriate. Chi-square test and Fisher exact test were used for comparing categorical variables. Receiver operator characteristic (ROC) curve analysis was undertaken to identify the value of the prediction score in predicting complication III–V. Multivariate analysis was performed using logistic regression. Level of P<0.05 were considered significant and all calculated P values were 2-sided.

## Result

### Study Population

A total of 1543 consecutive patients with HBV-related HCC were included in our study (Table 1). The majority of patients were with age <65 years (86.6%). 14.8% of the patients were with higher ASA grade (III and IV). 1543 liver resections included 397 segmentectomies (segment I:17, segment IV:44, segment V:76, segment VI:120, segment VII:92, segment VIII:48), 173 left lobectomies, 270 right hemihepatectomies, 146 left hemihepatectomies, 60 mesohepatectomies, 61 segmentectomies (VII and VIII), 61 segmentectomies (V and VI), 175 segmentectomies (VI and VII), 58 segmentectomies (V and VIII) and 142 other procedures. Portal hypertension was found in 27.7% of the patients. Major liver resection and extrahepatic procedures were respectively performed on 37.2% and 18% of the patients (Table 2).The top several extrahepatic procedures were adhesiolysis (5.6%), vascular procedures (4.0%), biliary tract exploration (2.5%), diaphragm resection (1.8%), bowel resection (1.2%) and splenectomy (1.0%). 34.9% of the liver resections were performed using the Hooking ligation technique. Based on intraoperative findings and pathological examination, 1430(92.7%) patients were with R0 resection, 77 (5.0%) with R1 resection and 36(2.3%) with R2 resection. 686 (44.5%) HCCs were within Milan criterion and the remaining 857 HCCs(55.5%) were out of Milan criterion. Based on postoperative pathological examination, the median Ishak score was 5 and 64.9% of the patients were with Ishak score more than 5. Overall postoperative morbidity rate was 30.1% (464/1543) and overall mortality rate was 1.5% (23/1543).The distribution of complication I–V was list in Table 3. Most common postoperative complications III–V were liver failure (21.6%), hydrothorax (20%), respiratory failure (12%) and biliary complication (16%).69.6% of the complications III–V were occurred in the hepatobiliary and respiratory system. The patients with grades III to V complications had longer hospital stays and ICU stays (P<0.001).

**Table 1:** Preoperative Characteristics of the patients.

Preoperative Characteristics	All patients	No Complications–	Grades III–V	P Value
	(n = 1543)	Grade II(n = 1418)	(n = 125)	
Male, (%)	1294(83.9%)	1183 (83.4%)	111 (88.8%)	0.118
Age≥65 years, (%)	207(13.4%)	186(13.1%)	21(16.8%)	0.247
BMI*(kg/m^2^), median (IQR*)	22.5(20.9–24.7)	22.6(20.9–24.7)	22.5(20.9–24.8)	0.620
HBsAg(+)*, (%)	1250(81%)	1150 (81.1%)	100 (80%)	0.764
HBeAg(+)*, (%)	246 (15.9%)	219(15.4%)	27 (21.6%)	0.072
HBV DNA(+)*, (%)	483(31.3%)	440 (31.1%)	43 (34.4%)	0.436
AST* (U/L), median (IQR*)	40(28–63)	39(28–62)	46(33–71)	0.005
ALT* (U/L), median (IQR*)	39(28–59)	39(28–58)	43(30.5–61.5)	0.104
Tumor size(cm), median(IQR)	5(4–8)	5(3.25–8)	5.75(4–8)	0.185
Tumor Number, (%)				0.241
Solitary	1311(85.0%)	1200(84.6%)	111(88.8%)	
Multiple	232(15.0%)	218(15.4%)	14(11.2%)	
Macrovascular Invasion, (%)	158(10.2%)	145(10.2%)	13(10.4%)	0.879
ASA*grade (III+IV), (%)	228(14.8%)	177(12.5%)	51(40.8%)	<0.001
Charlson index,median (IQR*)	1(0–1)	1(0–1)	1(0–1)	0.476
Hypertension, (%)	277(18.0%)	254(17.9%)	23(18.4%)	0.903
Pulmonary disease, (%)	47(3.0%)	39(2.8%)	8(6.4%)	0.049
Cardiovascular disease, (%)	42(2.7%)	40(2.8%)	2(1.6%)	0.605
Diabetes mellitus, (%)	117(7.6%)	105(7.4%)	12(9.6%)	0.377
Portal hypertension, (%)	428(27.7%)	345(24.3%)	83(66.4%)	<0.001

*HBsAg: hepatitis B surface antigen; HBeAg: hepatitis B e antigen; BMI:Body mass index.HBV-DNA(+) indicated hepatitis B DNA>2000 U/ml; IQR: interquartile range; ALT, alanine aminotransferase; AST, aspartate aminotransferase. ASA: American Society of Anesthesiologists category. Pulmonary disease indicated chronic obstructive pulmonary disease, asthma, chronic bronchitis and tuberculosis. Cardiovascular disease included coronary heart disease, previous coronary revascularization, cerebral arterial occlusive disease, and/or peripheral vascular occlusive disease. Tumor size indicated the sum of all the tumors diameter.

**Table 2:** Intraoperative Parameters and Postoperative Outcomes.

Intraoperative Parameters	All patients (n = 1543)	No Complications–Grade II(n = 1418)	Grades III–V (n = 125)	P value
Major resection, (%)	574 (37.2%)	489(34.5%)	85(68%)	<0.001
Extrahepatic procedures,(%)	278 (18%)	236(16.6%)	42(33.6%)	<0.001
Diaphragm resection	28(1.8%)	24(1.7%)	4(3.2%)	0.389
Vascular procedures	61(4.0%)	47(3.3%)	14(11.2%)	<0.001
Splenectomy	16(1.0%)	11(0.8%)	5(4.0%)	0.003
Bowel resection	18(1.2%)	14(1.0%)	4(3.2%)	0.076
Biliary tract procedures	39(2.5%)	32(2.3%)	7(5.6%)	0.047
Inflow occlusion, (%)	642(41.6%)	592(41.7%)	50(40.0%)	0.389
Hooking with ligation,(%)	538 (34.9%)	501 (35.3%)	37 (29.6%)	0.205
Ultrasonic dissector,(%)	1005(65.1%)	917(64.7%)	88(70.4%)	0.205
Blood loss (ml), median (IQR*)	400(200–600)	400(200–530)	500(300–800)	<0.001
Blood transfusion, (%)	288 (19.3%)	244(17.2%)	44 (35.2%)	<0.001
ICU*stay(days), median (IQR*)	0(0–1)	0(0–1)	1(0–2)	<0.001
Hospital stays(days), median (IQR*)	12(10–15)	12(10–14)	19(13–24)	<0.001

*ICU: Intensive Care Unit. IQR: interquartile range. Vascular procedures included resection tumor thrombus of portal vein, hepatic vein and procedures including vena veins.

**Table 3:** Postoperative Complications Grade.

Postoperative Complication Grade, Number (%)	All patients (n = 1543)	No Complications–Grade II(n = 1418)	Grades III–V (n = 125)
No complications	1079(69.9%)	1079(76.1%)	0
Grade I	146(9.5%)	146(10.3%)	0
Grade II	193(12.5%)	193(13.6%)	0
Grade IIIa	58(3.8%)	0	58(46.4%)
Grade IIIb	13(0.8%)	0	13(10.4%)
Grade IV	31(2%)	0	31(24.8%)
Grade V	23(1.5%)	0	23(18.4%)

### Development of the Prediction Score

Univariate analysis (Table 1 and Table 2) between complications 0–II group and complications III–V group showed four preoperative factors and five predictable intraoperative factors were significantly different. They were ASA grade, pulmonary disease, portal hypertension status, AST level, extrahepatic procedures, biliary tract procedures, splenectomy, vascular procedures and extent of liver resection. Considering the three extrahepatic procedures (biliary tract procedures, splenectomy and vascular procedures) may significantly influence the postoperative outcomes, they were also included to develop the prediction model. Though the three factors of extrahepatic procedures (including biliary tract procedures, splenectomy and vascular procedures) and extent of liver resection were intraoperative factors, they were still considered as predictive factors because they usually could be predicted by imageology examination before surgery.

**Figure 1:**
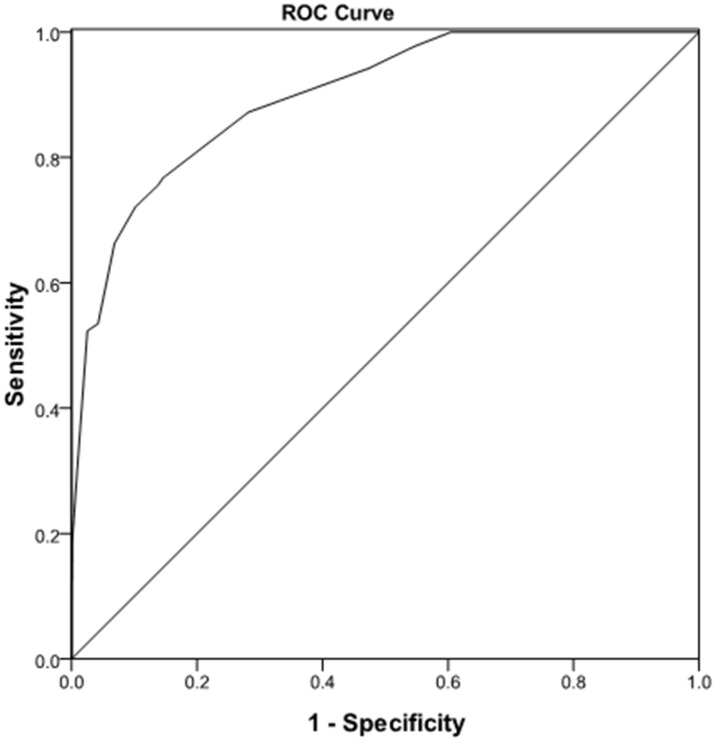
Receiver operator characteristic curve analysis of preoperative risk factors to predict liver resection postoperative severe complication in development group (area under the curve  = 0.898,P<0.0001).

70% (1080/1543) of the cohort were randomly selected as derivation population to develop the prediction model and the remaining 30% patients (463) were evaluated for the score validation. The above nine risk factors were selected for inclusion in the logistic regression analysis (Table 4) and the results showed that extent of liver resection, portal hypertension, ASA grade and extrahepatic procedure were significantly different (P<0.001).Major resection showed the greatest association (OR = 23.473) with complications grade III to V, followed by portal hypertension(OR = 20.502), ASA(OR = 7.999) and extrahepatic procedure(OR = 3.503). The point score ranged from 0 to 4 for individual predictor and points were summed up (ranging from 0 to 10 points). The total scores were grouped into three groups (score 0–3 for low, score 4–6 for moderate and score 7–10 for high risk group) based on clinically relevant estimated complication III–V risks. The incidence of postoperative complication III–V was 65.6%, 11.9% and 1.6% for high, moderate and low risk group, respectively. The high risk means that a patient with a preoperative score between 7 and 10 had 65.6% chance to develop complications grade III to V. Receiver operator characteristic (ROC) curve (Figure 1) analysis with an area under the ROC curve of 0.898(P<0.0001) revealed that the prediction score identified patients at significant risk for development grades III to V complications.

**Table 4:** Development of Predictor Score Based on Logistic Regression Analysis.

Predictor	Categories	Regression Coefficient β	Odds Ratio (β)	p value	Risk Score
Resection Extent	Major	3.156	23.473	<0.001	4
	Minor	Reference	1		0
Portal hypertension	Yes	3.021	20.502	<0.001	3
	No	Reference	1		0
ASA	III and IV	2.079	7.999	<0.001	2
	I and II	Reference	1		0
Extrahepatic procedure	Yes	1.254	3.503	<0.001	1
	No	Reference	1		0

### Validation of the Prediction Model

**Figure 2:**
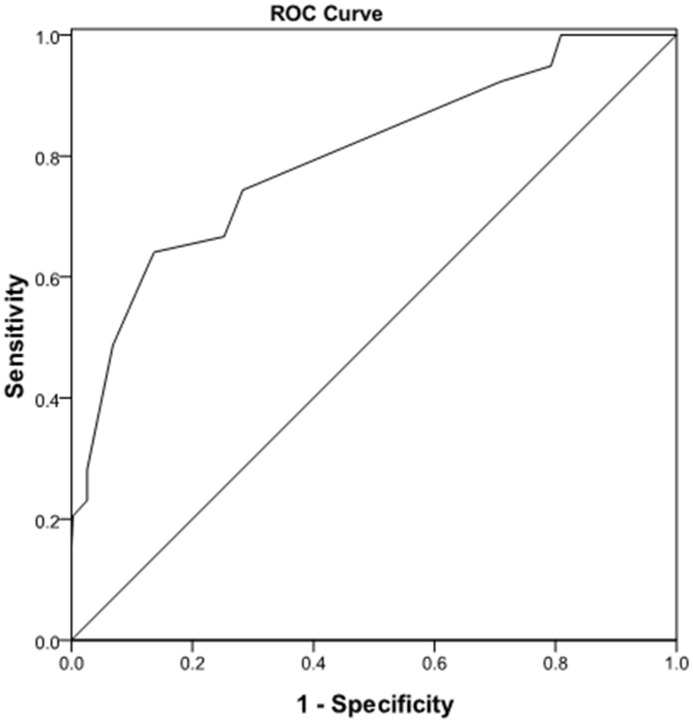
Receiver operator characteristic curve analysis of preoperative risk factors to predict liver resection postoperative severe complication in validation group (area under the curve  = 0.798,P<0.0001).

The remaining 463 (30%) patients were used for the validation of the prediction model. Based on the logistic model, the predicted mean risk for complications III–V was 10.4% (48/463), whereas the observed risk was 8.4% (39/463). The overall predicted risk differed from the observed risk by only 2%. Predicted and observed mean risks were very similar across the entire range of risk for grades III to V which was confirmed by the Hosmer-Lemeshow test (P = 0.466). Receiver operator characteristic (ROC) curve (Figure 2) analysis revealed an area under the ROC curve of 0.798(P<0.0001).

## Discussion

In the case of HCC, the tumor usually arises in a liver with some degree of cirrhosis, which is a contributor to worse outcome for any major procedure [2], especially in China where a high prevalence of HBV carriage still existed. Despite decidedly lower mortality rates with less than 4%, the incidence of postoperative complications for HCC patients undergoing liver resection remains high. When comparing complication to other surgery centers, the most difficulty is the lack of consensus on how to define complications and to stratify them by severity. The Clavien-Dindo complications classification system [22], mostly relying on the therapy, provides a standardized and reproducible tool to allow comparison among different centers with different therapy strategy. We herein presented the surgical outcome of a high volume cohort with HBV-related HCC resection according to the Clavien classification system.

The mortality rate in our study was 1.5%, which was lower than the 3.15% in a meta-analysis including 35,000 hepatic resections [3]. In our study, all the liver resections were performed in the recent several years (from 2009 to 2013) when surgical techniques and perioperative management have obviously improved, which could explain the difference. Although, all the liver resections were performed when the CTP class was A, 52% of death was caused by liver failure (12/23), probably owing to the damaged hepatic parenchyma of HBV and better function of other organs. With an overall 30.1% morbidity rate, our results were consistent with those reported in recent studies [1], [3], [6], [7], [8], [9], [10]. Death was rare in our cohort, therefore it was not analyzed alone and combined with other lethal complication(complication III–IV).

To the present, efforts to assess patients' postoperative complications have almost involved in all the perioperative parameters, such as extent of resection, blood loss, operation time and so on. These risk factors are associated with complications and can help surgeons postoperatively take necessary measure to avoid risk. However, there are rare exclusively parameters limited to preoperative course and provide postoperative risk stratification for both surgeons and patients to determine patients' eligibility for surgery [29], such as the preoperative clinical risk scores of CTP and the MELD score. The two scores are widely used in surgery centers for preoperative evaluation, but they only reflect the liver function.

The present study represented an important step in quantifying and predicting risk of postoperative severe complications. Based on preoperative and predictable intraoperative parameters, logistic regression analysis revealed 4 clinical parameters as predictors of perioperative morbidity. They were major liver resection, portal hypertension, ASA grade and extrahepatic procedure, and they performed well in its discriminatory ability with a high accuracy given the ROC AUC of 0.898. For clinical usability, we integrated the four parameters in a simple and intuitive prediction score that can be determined before surgery to guide treatment decisions. The score system showed excellent discrimination of postoperative complication severity (mild, moderate, high) among 3 risk groups, with the risk rate 1.6%, 11.9% and 65.6%, respectively.

In our prediction model, the parameters ASA score and portal hypertension were easily obtained in the preoperative period and the two other parameters just needed routine assessment on CT or MRI. Each parameter reflected a different preoperative status of the patient. The ASA score reflected the whole eligibility of patients for surgery. Many demographic characteristic parameters, such as age [30], comorbidities [24], [25] (such as diabetes or cardiovascular), have been identified as risk factors for postoperative mortality or morbidity. But they were not considered for the regression model because of the lack of statistical effect at univariate analysis and partly taken into account by the ASA score.

Resection extent and extrahepatic procedures reflected the operation related damage degree to patient. Patient who underwent resection of more than 3 segments or an additional extrahepatic procedure had increased complications. The two factors were independent predictors of postoperative complications III–V in our study and also were verified by many other studies [1], [6], [10].

The portal hypertension represented the underlying damage of liver parenchyma and was an independent predictor of postoperative complications in our study. Liver parenchyma impairment included fibrosis, cirrhosis and liver steatosis. HBV related-HCC always arises in a liver with some degree of cirrhosis in China. In our study, 64.9% of the patients were cirrhosis (Ishak score more than 5) based on postoperative pathological examination, however, the fibrosis degree could not be predicted before operation. Therefore, we selected portal hypertension as the representing of liver parenchyma of HBV infection patients because some studies suggested that an increased portal hypertension was associated with worse underlying liver damage [26], [31], [32], [33]. In addition, liver steatosis is one risk factor for postoperative liver dysfunction and preoperative evaluation of steatosis is important for prediction complication. BMI have been proved to accurately identify the grade of hepatic steatosis [34], [35] and univariate analysis of BMI showed no differences in our study.

We did not consider the biochemical parameters reflecting liver function such as bilirubin, albumin, ascites and prothrombin time that were taken into account in the CTP score, because all the patients were operated with a CTP class A. By univariate analysis, AST level in complication III–V group was significantly higher than that of the patients in group with complication less than II. Increased AST levels reflected the presence of potential underlying liver disease or liver damage and it did not reach statistical significance when analyzed in logistic regression analysis. In addition, HBsAg and HBeAg were not different between the two groups (P = 0.764, P = 0.072, respectively), although HBV serum markers level are associated with fibrosis severity [36], [37], [38].A reasonable interpretation may be that the three parameters together reflected the liver parenchyma injury and were included in the parameter of portal hypertension. The other parameters, biliary tract procedures, splenectomy and vascular procedures, were significantly different between complications 0–II group and complications III–V group, however, they were not independent risk factors for postoperative severe complications. That is because they only reflected one side of the extrahepatic procedure. Although some other factors, such as blood loss and transfusion, were associated with complications, they were not included in our model because these parameters could not be predicted before operation and did not contribute to planning the operation.

Several studies have developed simple scores to predict the risk of complications in liver resection depending on perioperative parameters like ours, such as ASA grade, extrahepatic procedure and extent of liver resection. But, many of these scores included intraoperative factors such as blood loss and operation time, these parameters were not predictable and the scores could not be used for preoperative patient selection. Although Breitenstein [1] and Andres [6] developed scores integrating only preoperative factors to predict the risk of complication, they also included benign disease and metastatic hepatic carcinoma except for HCC. As far as we know, we developed the first preoperative score for predicting complications on HBV-related HCC patients. Our results turned out that HBV-ralated HCC patients had a specific preoperative score for predicting complications. In addition, we firstly selected the factor of portal hypertension to reflect the degree of underlying liver damage in a model.

We retrospectively analyzed the relationship between severe complications and preoperative and predictable intraoperative parameters in high-volume surgical center. Based on preoperative and predictable intraoperative parameters, this score represented a stratification of the risk of postoperative complication in HBV-related HCC patients undergoing liver resection. This prediction scoring systems have been proposed to improve patient selection and is helpful to identify patients at high risk of complication III–V in future. To reduce the incidence of postoperative severe complications, careful preoperative planning and better patient selection are required. Using the prediction score, for patient with a score of more than 7, the operation scheme is advised to modify because incidence of postoperative complication III–V was 65.6%. For a score of less than 3, the operation should be taken decisively. But for a score of 4–6, sufficient preoperative management and informed consent to patients are necessary.

The results of our study should be interpreted cautiously because this prediction score is the absence of validation in other liver resection centers. Moreover, our analysis was restricted to patients with HBV-related HCC, this may be not appropriate for liver resection for other reasons. Third, we only included preoperative and predictable intraoperative factors in our model for preoperative patient assessment and selection, and others factors, such as fibrosis severity and blood loss were not included. So, the model would not suitable for postoperative management.

In conclusion, based on four preoperative risk factors, we developed and validated an integer-based risk score to predict postoperative severe complication after liver resection for HBV- related HCC patients. This score allows identifying patients at high risk and may be contribute to preoperative risk stratification and clinical decision-making.
